# Radiation-Driven Polymerisation of Methacrylic Acid in Aqueous Solution: A Chemical Events Monte Carlo Study

**DOI:** 10.3390/gels9120947

**Published:** 2023-12-01

**Authors:** Aleksandras Sevcik, Zilvinas Rinkevicius, Diana Adliene

**Affiliations:** 1 Department of Physics, Kaunas University of Technology, Studentu 50, 51368 Kaunas, Lithuania; rinkevic@kth.se; 2 Department of Theoretical Chemistry and Biology, KTH Royal Institute of Technology, Brinellvägen 8, 11428 Stockholm, Sweden

**Keywords:** Monte Carlo, radiation-induced polymerisation, methacrylic acid, hydrogels, polymer chemistry, micro-structure simulation, growth pattern

## Abstract

This study employed a coarse-grained Monte Carlo (MC) simulation to investigate the radiation-induced polymerisation of methacrylic acid (MAA) in an aqueous solution. This method provides an alternative to traditional kinetic models, enabling a detailed examination of the micro-structure and growth patterns of MAA polymers, which are often not captured in other approaches. In this work, we generated multiple clones of a simulation box, each containing a specific chemical composition. In these simulations, every coarse-grained (CG) bead represents an entire monomer. The growth function, defined by the chemical behaviour of interacting substances, was determined through repeated random sampling. This approach allowed us to simulate the complex process of radiation-induced polymerisation, enhancing our understanding of the formation of poly(methacrylic acid) hydrogels at a microscopic level; while Monte Carlo simulations have been applied in various contexts of polymerisation, this study’s specific approach to modelling the radiation-induced polymerisation of MAA in an aqueous environment, utilising the data obtained by quantum chemistry modelling, with an emphasis on micro-structural growth, has not been extensively explored in existing studies. This understanding is important for advancing the synthesis of these hydrogels, which have potential applications in diverse fields such as materials science and medicine.

## 1. Introduction

Radiation-induced polymerisation is a process in which ionising radiation is used to initiate chemical reactions in monomers, leading to the growth and formation of the new polymers. There are three main steps of polymerisation reactions: (a) initiation, where radicals are formed due to the breakage of chemical bonds in monomer molecules; (b) propagation, where the polymer chain grows and forms a cross-linked network; and (c) termination, which encompasses several reactions, where radicals are consumed or combined with other radicals [1,2]. The understanding of radiation-induced polymer growth mechanics is primarily utilised in the context of 3D dosimeters, which involve the use of monomers dissolved in water or integrated into hydrogel compositions. When radiation-induced polymerisation proceeds, more dense polymerised areas in the gels are created, which accurately record the 3D distribution of the radiation dose with high spatial resolution and precision [3,4]. Radiation-sensitive gels can be explored in medical and pharmaceutical applications. For example, these gels can be used as drug-delivery systems, where the release of a drug can be triggered by ionising radiation [5]. They have also been used in tissue engineering, promoting the growth of cells or tissues as a response to the applied ionising radiation [6,7,8]. The use of ionising radiation is also important in the synthesis of polymer-based nanomaterials, such as nanoparticles or nanocomposites, as it enables the production of materials with specific structures and specific properties for different applications [9,10].

Modelling plays a crucial role in polymer reaction engineering, as it allows for better process control, the prediction of the molecular structure, and the tuning of the properties. There are two main groups of polymerisation modelling methods: statistical and deterministic [11]. Deterministic methods are based on mass balance or rate equations and describe the concentration variations of different species over the time [12,13]. These methods can be used to predict the evolution of a polymerisation reaction by solving the equations numerically. The other type of deterministic method is the molecular dynamics (MD) method [14]. This involves the integration of Newton’s equations of motion for each atom in the system and allows for the prediction of the positions and velocities of the atoms at any given time.

The Monte Carlo (MC) method is a type of statistical modelling technique that relies on the generation of a random number of events, which are used for the simulation of a system’s behaviour over time. These methods are widely used in polymer engineering for the simulation of a wide range of characteristics of polymers, such as the chain length, branching, monomer sequence, functionality, and cross-linking density [15,16,17,18,19]. One of the key advantages of the Monte Carlo method is its ability to provide information about the polymer micro-structure, which is impossible using other modelling techniques. It can be applied to polymerisation reactions, in which monomer propagation or chain transfer events can be simulated, primarily through the use of coarse-grained model assumptions, which eliminate the microscopic degrees of freedom and represent a polymer through a simplified structure [20,21]. However, the stochastic nature of MC simulations means that they can be computationally intensive and time-consuming, especially for large-scale or highly detailed simulations. This can limit their practical applicability in cases where quick and efficient modelling is required. In addition, they may struggle with accurately capturing long-range interactions and the subtle nuances of polymer dynamics, potentially leading to less-precise predictions of the properties and behaviours of polymers. Finally, the assumptions of coarse-grained models, which simplify the polymer structures by eliminating the microscopic degrees of freedom, can lead to a loss of detailed information about the polymer’s physical and chemical properties. This simplification may result in inaccuracies when predicting the behaviour of more-complex polymer systems.

MC simulations are particularly well suited to the simulation of radical polymerisation, in which the polymerisation occurs due to the free radicals introduced in the system [22,23]. In this approach, the radical-based polymerisation process is divided into three stages: the nucleation of radicals, the growth of chains within a solvent of monomers, and termination. However, in order to simulate radiation-induced polymerisation, a multi-scale modelling approach is required: first, the simulation of radiation transport to the media should be performed, and then, in the next stage, the MC simulation of radical-like polymerisation can proceed.

In this paper, we present the coarse-grained MC approach of radiation-induced polymerisation of MAA in an aqueous solution, where the CG bead is equal to a whole monomer, and the external radiation is introduced to form the random radicals. The focus of this research was to analyse the micro-structure and growth pattern of the of the radiation-induced polymerisation of MAA in the aqueous solution, which would allow optimising the the synthesis of poly(methacrylic acid) hydrogels with unique properties [1,24,25].

## 2. Results and Discussion

### 2.1. Results

The radiation-induced growth of MAA polymers is a complex chemical process involving multiple competing chemical reactions and typically leads to the creation of simple and branched MAA polymers. The outcome of this process is stochastic in nature and, on the individual MAA polymer level, is driven to a large extent by unique chemical events happening during the growth of specific MAA polymers. This chemical process could not be easily simulated by chemical kinetics methods, as these methods have limited capabilities to describe the growth of MAA polymers beyond the statistically averaged picture. To overcome these limitations of chemical kinetics methods, we employed the stochastic Monte Carlo method, which allowed us to describe the growth of MAA polymers on individual and collective levels. In this work, to study the radiation-induced growth of MAA polymers, we carried out Monte Carlo simulations using the setup described in the computational details section and repeated these simulations on 70 randomly generated initial simulation boxes.

**Figure 1:**
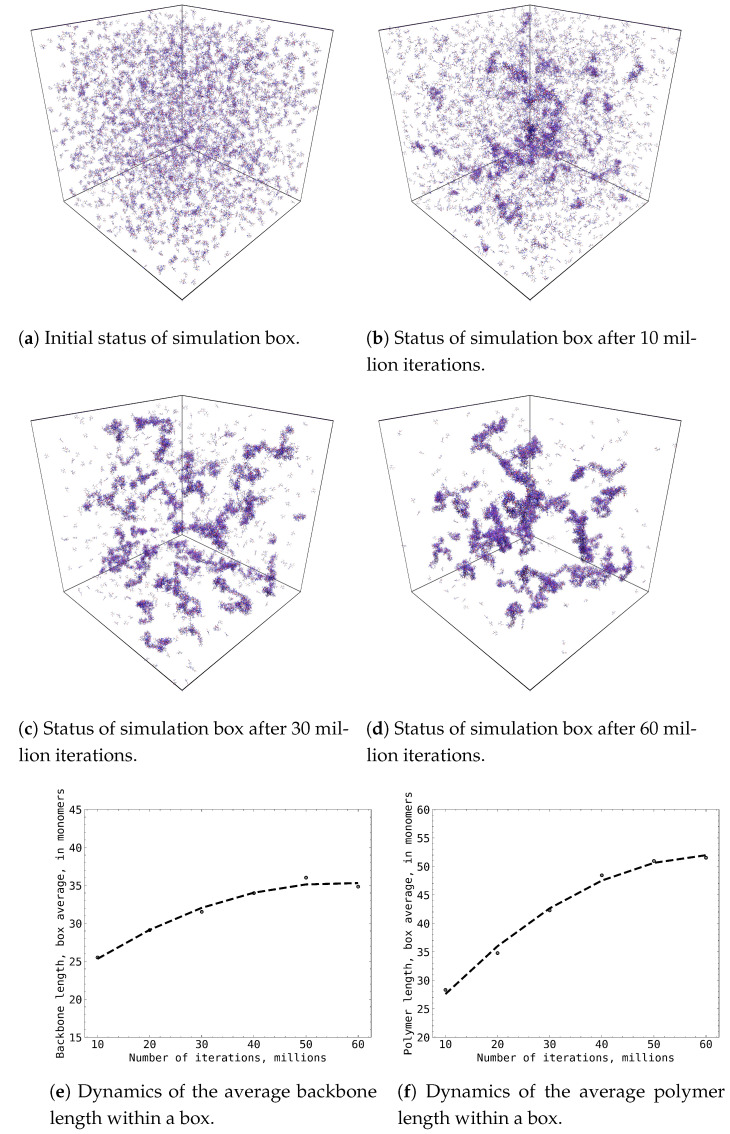
The growth of MAA polymers during Monte Carlo simulation: (**a**–**d**) subplots represent evolution of 50 × 50 × 50 nm simulation box with 5875 MAA molecules at different stages of simulation; (**e**) subplot depicts evolution of averaged MAA polymer backbone length (in MAA monomers) during simulation; (**f**) subplot depicts evolution of averaged MAA polymer length (in MAA monomers) during simulation.

In each Monte Carlo simulation, 60 million random walk steps in the chemical events space had been executed to emulate the growth of MAA oligomers, referred to as polymers for simplification, under a dose rate of 20 cGy/min of γ-radiation. In all 70 cases, we observed the formation of complex branched MAA polymers with a distinct grouping of smaller branched polymers into an agglomeration of MAA polymers. A typical MAA polymer growth pattern in our Monte Carlo simulations is displayed in Figure 1, where the initial formation of MAA polymer agglomeration centres was already observed after 10 million Monte Carlo steps, and consolidation of initial branched polymers continued up to 50 million Monte Carlo steps, where a relatively stable agglomeration of MAA polymers was reached. Across all the simulations, we observed the formation of intricate branched MAA polymers, with smaller branched polymers aggregating to form larger clusters of MAA polymers. The average length of the polymer backbone reached approximately 35 monomers, exhibiting fluctuations between 25 and 50. Similarly, the average polymer length reached around 50, with fluctuations ranging from 38 to 72 monomers.

**Figure 2:**
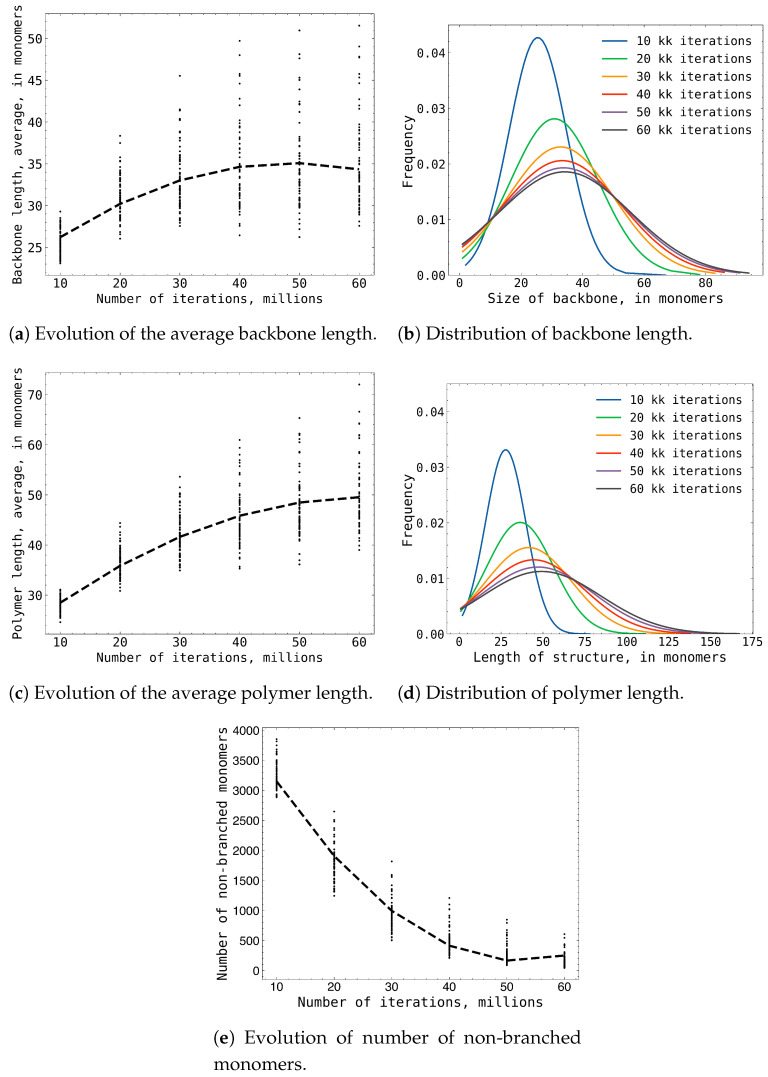
The combined results of the 70 box simulation statistics: (**a**,**b**) subplots shows the evolution and distribution of the average backbone length; (**c**,**d**) subplot shows the evolution and distribution of the average polymer length; (**e**) subplot shows the number of non branched monomers during the simulation.

In Figure 2, we present the statistical analysis of MAA polymer growth from our Monte Carlo simulations. These plots show that the growth of MAA polymers continued up to 50 million Monte Carlo steps. At this point, a state of equilibrium was reached between the growth of MAA polymers and the degradation of branched MAA polymers. This equilibrium resulted in a stable structure of MAA polymers within the simulation box. However, the structure of MAA polymers was not static; it continued to evolve rapidly, influenced by the ongoing balance between the formation and breakdown of MAA polymers. This dynamic process was clearly demonstrated in plot Figure 2e, where the continuous changes in the polymer structure could be observed.

The outlined trends in the growth of MAA polymers were statistically convergent, as extending the simulation sample of simulation boxes from 70 to 420 did not change the results presented in Figure 2.

**Figure 3:**
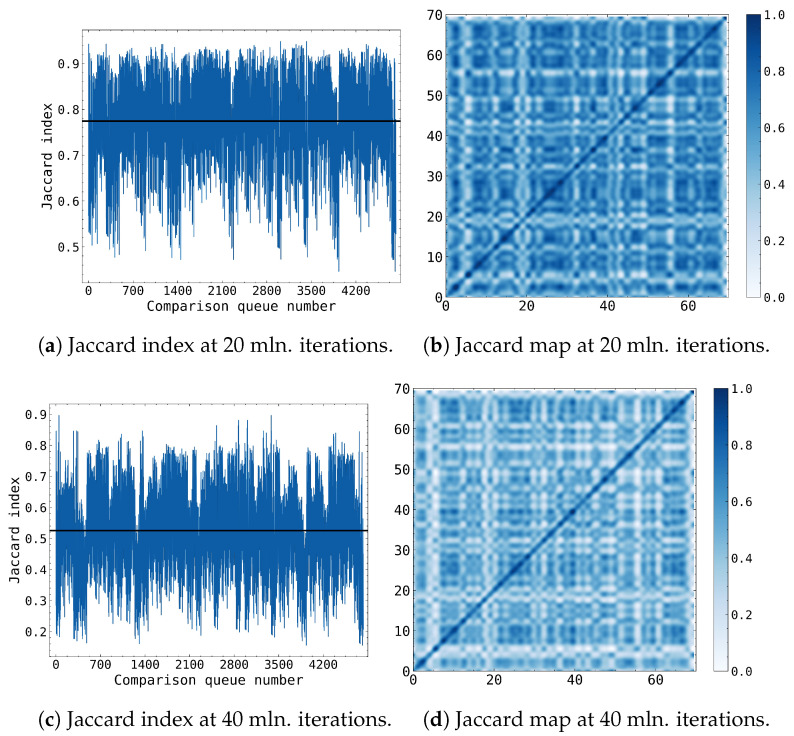
Jaccard similarity index distributions and maps at various simulation phases. Each of the 70 boxes had been compared against each other and the Jaccard index had been calculated, thus resulting in 4900 comparisons that had been set on the x-axis. (**a**,**b**) at 20 million iterations showing the very high similarity between the polymers formed in the boxes; (**c**,**d**) at 40 million iterations showing decreasing similarity between the polymers formed in the boxes.

Our Monte Carlo simulations effectively generated distinct MAA polymer growth patterns in each simulation box, enabling the investigation of the variability of MAA polymer growth, including rare growth events. Analysing MAA polymer growth patterns across various simulation boxes aided in understanding the primary trends of MAA polymer growth and identifying the key chemical processes that facilitated MAA polymer formation under γ-irradiation. In Monte Carlo simulations, evaluating statistical randomness is an essential aspect to ensure the accuracy and reliability of the results. We employed the Jaccard similarity index to assess the resemblance of growth patterns among different simulation boxes (Figure 3). The Jaccard index is a similarity statistic that compares the size of the intersection of two sets to the size of their union in order to determine their similarity. It can be used to quantify uncertainty in Monte Carlo simulations by comparing the sets of outputs obtained from different simulation runs. Additionally, it quantifies the level of unpredictability in the simulation results by providing a measure of consistency or resemblance between the sets. The Jaccard index values and simulation outcome maps demonstrated consistent results, capturing the anticipated variability in polymer growth due to the inherent randomness of the MC method. After 20 million iterations, a high degree of similarity between boxes was observed in Figure 3a, suggesting that the simulation parameters and conditions ensured consistent outcomes across multiple runs, despite the random sampling inherent in the Monte Carlo approach. However, as the simulation progressed, the similarity diminished as expected (Figure 3c), due to the formation of more complex structures from increasingly intricate combination events with lower probabilities, resulting in greater variability in the final state of the box. Extending the Jaccard similarity analysis from 70 to 420 simulation boxes did not significantly alter the similarity index values among the boxes. Consequently, these findings affirmed that our MC simulations were statistically convergent and accurately represented the dominant pattern of MAA polymer growth in our model.

### 2.2. Discussion

A review of Monte Carlo simulations of polymers showed a diverse range of coarse-grained models, each uniquely tailored to specific research objectives [26,27,28,29]. However, due to the inherent differences in their setups and purposes, a direct comparison with our study’s methodology and goals was not feasible. Our study’s MC simulations, instrumental in exploring the polymerisation of MAA in an aqueous solution, were grounded in a sophisticated approach combining detailed micro-structural insights with advanced quantum mechanical methods. Each simulation step initiated with the generation of a radical, with its interaction with monomers determining the ensuing growth or decomposition reaction. This process, governed by calculated probabilities and augmented by random rotations and translations of monomers and oligomers, was meticulously checked for physical feasibility. Any detected collision led to the reversal of the simulation step, maintaining the system’s integrity. In this initial version of our novel approach, the model was specifically designed for simulations with a standard gamma irradiation dose rate of 20 cGy/min, using only water as the solvent and methacrylic acid as the molecule. Due to the complexity of the involved mechanisms, such limitations were necessary for feasibility. Future versions aim to broaden these parameters, enhancing the model’s scope and applicability.

A cornerstone of our approach was the quantum mechanical accuracy in calculating bond dissociation and radical formation rates. Employing sophisticated quantum chemical models, specifically the hybrid B3LYP/def2-SVPD approach, we optimised the geometry of each MAA molecular system. This process of geometry optimisation aimed to find the most stable configuration of the molecule by minimising total energy. The def2-SVPD basis set, part of the Karlsruhe series, balanced computational efficiency with the accuracy needed for complex calculations.

Our MC-part methodology was based on a simplified model that emphasised combination and breakdown reactions (see Supplementary Materials for the main pseudocode). This strategic choice addressed the need for computational efficiency and provided clarity in theoretical understanding, essential in the complex realm of polymerisation; while this simplification allowed for the extraction of fundamental insights into polymer growth, it also necessitated several assumptions and boundary conditions. The simulation began in a controlled environment containing only monomers, without pre-existing polymers or radicals, and operated within a fixed geometric boundary. The methacrylic acid molecule was geometrically simplified for computational manageability. These assumptions, while facilitating the observation of polymer growth, also limited the representation of real-world conditions. Despite the rigorous quantum mechanical methods and the careful considerations in model construction, our simulation simplified several aspects of physical and chemical interactions. It did not fully account for complex secondary reactions, solvent and oxygen effects, diffusion limitations, and spatial effects in real polymerisation. Moreover, the model implicitly accounted for termination reactions in free radical polymerisation but overlooked disproportionation termination events, among other secondary reactions.

Direct experimental verification in our study encountered several challenges, including issues related to solvent compatibility, relaxation effects, accurate molecular weight determination, signal overlap, sensitivity, and stringent sample requirements. Tackling these challenges would necessitate thorough sample preparation, fine-tuning of experimental parameters, and possibly the integration of Nuclear Magnetic Resonance (NMR) with other analytical techniques. Given these experiments’ inherent complexity and high uncertainty, computational models are valuable in polymer science, helping to circumvent some of the experimental methods’ limitations. While our study did not include direct experimental validation, it found a nuanced parallel with certain specific conditions in Pulsed-Laser Polymerisation (PLP) studies related to MAA polymerisation [30,31]. It is important to note that these parallels should not be interpreted as direct evidence but rather as an indicative correlation under particular circumstances. This comparison, while not exact, suggested that the oligomer sizes observed in our model were within a plausible range, reinforcing the potential validity of our theoretical approach.

Acknowledging these simplifications and assumptions is crucial in interpreting the results of our MC simulations; while the model offered a robust framework for understanding fundamental polymerisation processes and had undergone rigorous mathematical validation on a solid modelling base, including passing uncertainty checks, it did not capture the complete complexity of real-world polymerisation dynamics. Nevertheless, the understanding derived from this model could be beneficial for studying the free radical-induced polymerisation process.

## 3. Conclusions

This study utilised MC simulations, augmented by quantum mechanical modelling, to investigate the polymerisation of MAA in aqueous solutions under radiation-induced conditions. The use of quantum chemistry data from a B3LYP/def2-SVPD model enabled a detailed analysis of polymer growth. The structural output concerning polymer length and counts, as derived from our simulations, facilitated the quantification of the degree of polymerisation, polymer molecular mass, and polydispersity.

The findings contributed to a more comprehensive understanding of the structural dynamics of MAA polymers. However, it was acknowledged that the simulation model, while providing valuable insights, might not fully encapsulate all aspects of polymer behaviour under varying real-world conditions. Further research is warranted to expand upon these findings, potentially incorporating broader data sets and exploring alternative simulation methodologies. The model’s adaptability and capacity to be adjusted makes it a great resource for the study of polymer growth and the formation of polymers. With further development, it could facilitate the design and synthesis of polymers with tailored properties, as well as enable the exploration of various scenarios and optimisation of conditions for polymer growth.

## 4. Materials and Methods

### 4.1. Chemical Event Monte Carlo Model: Implementation Details

**Figure 4:**
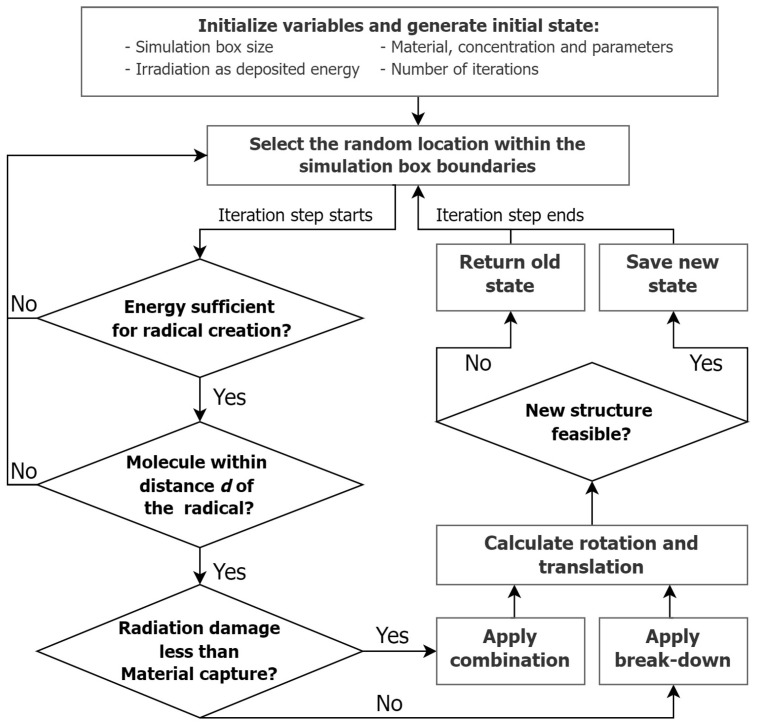
Code flowchart of MC cycle. In the simulation, a box of material and its concentration were initiated. A random location within the box was selected, and energy was checked to ascertain if it could generate a radical. If a molecule lay within range, energy calculations were made for radiation damage and material capture. Three outcomes were possible: a changed molecular structure, an unchanged structure, or no event. In the first two scenarios, the new coordinates were calculated, and a feasibility check was performed. If the changes were deemed physically possible, the simulation box was updated.

The model was based on the MC method, which relies on repeated random sampling to obtain numerical results, as polymer growth upon irradiation can be described by a probabilistic interpretation. The simulation box of the desired size was initiated and filled with the type of material of interest and its concentration, i.e., the number of molecules of the material per the volume of the box, as well as their initial configuration, and the MC cycle was started (Figure 4). The iteration step started with the selection of a random location within the box’s geometrical boundaries, which simulated the possibility of the random appearance of radicals. A check of the energy within that box location was performed to ensure that the absorbed energy value was sufficient to create a radical. The next step checked if there was a molecule of interest within a certain distance that would be affected by the creation of the radical. If this were the case, the energy calculation for radiation damage and material capture was performed in order to set the possible combination or break-down event sequence depending on those energy calculations. At this stage, there were three potential scenarios: a changed molecular structure following a successful combination event, a changed molecular structure resulting from a successful break-down event, or an unchanged structure if neither of these events occurred successfully. In all cases, the next step was the calculation of rotation and translation, followed by a final feasibility check that verified that the changes were physically possible, i.e., that there was no unacceptable intersection of the simulated bodies. The result of the check determined, accordingly, whether the changes made were retained and the simulation box was updated or whether they were reversed.

The initial simulation box geometrically bounded the simulation volume and generated mononomers, dimers, and trimers in roughly the same amounts at the default concentration. The bond angle was equal to 109.5°, corresponding to the bond angles of carbon atoms. The structure could be subjected to Break-down and Rotation–Translation, Combination and Rotation–Translation, or solely to Rotation–Translation, based solely on the computations. The Rotation–Translation event calculated the moment of inertia of the specified structure and created random rotations and translations based on this value. The object was then rotated and translated along the X, Y, and Z axes by a random amount. The maximum distance allowed was no greater than six times the length of the longest edge of the molecule.

The breakdown event forced the structure of interest to segment off into new distinct objects at the event point. The combination event implemented the possible scenarios of connection of the structures when the activated molecule was within a certain distance from the other surrounding bodies. The selection of the exact Combination scenario depended on the configurations, locations, and lengths of the structures of interest. The objects might merge by interconnecting within the new entity, hence expanding its size. When both or one of the objects were small in size, or when the activation point was close to the end, the shorter object joined the longer object, and the chain grew. In the case of larger, complicated, branched structures, the combination via the activation point could produce the new interconnected structure. In other scenarios, an object could become a branch of another object and create a branched structure. Depending on its size and activation point, the object might either produce a single branch or split into two branches at the activation point. Other combination scenarios included complicated swaps where the activated structures might break down at specific spots and join the split portion of another structure, thus creating new structures with new connections and relationships.

The feasibility check consisted of the identification of collisions and was required for the control and rejection of physically implausible outputs generated by a random engine. In contrast to the detection of sphere collisions, the detection of cuboids necessitated a more complex method, which had to be as efficient as feasible owing to the high number of cuboids and the required number of iterations. The Gilbert–Johnson–Keerthi (GJK) distance method is a technique for calculating the shortest distance between two convex sets [32]. The class itself had been adapted from the OpenGJK library [33], which includes the fastest and most accurate version of the GJK algorithm available to date. The project also employed the vector type Mersenne Twister pseudorandom number generator (MT19937) from Intel’s Math Kernel Library [34] to produce a uniform discrete sample distribution with the generator’s period of $2^{19,937^{-1}}$.

### 4.2. Chemical events Monte Carlo Model: Parameterisation

The initial parameters for the study were established using simulations based on Density Functional Theory (DFT). In these simulations, the hybrid B3LYP/def2-SVPD approach was employed for the geometry optimisation of each molecular system, specifically those of MAA polymers with varying chain lengths (n = 1 to 30). The B3LYP component of this hybrid functional merged Hartree–Fock exchange with DFT principles to enhance calculation precision. The def2-SVPD term referred to a chosen basis set in quantum chemistry calculations, integral for constructing molecular orbitals. The def2 designation indicated the Karlsruhe series basis sets, and SVPD represented Split Valence with Polarisation and Diffuse functions. This basis set was selected for its effective balance between computational efficiency and the accuracy required for complex quantum chemical calculations [35]. Geometry optimisation involved iteratively adjusting atomic positions to ascertain the most energetically favourable molecular configuration. This process sought the lowest possible energy state for the molecule, ensuring that the optimised geometry formed the cornerstone for subsequent analyses. The accurate determination of this optimised structure was crucial, as it underpinned further evaluations of the molecular system’s stability and potential chemical reactivity.

Upon the completion of geometry optimisation, the study proceeded to calculate the bond dissociation and radical formation energies in the MAA molecules. This task was carried out using the quantum mechanical method B3LYP/def2-TZVPP [36]. The method included triple-zeta valence polarisation functions, crucial for addressing electron correlation effects, and diffuse functions, which were essential for capturing the electron density in peripheral regions of the molecule. For the series of molecules denoted as (MAA)n, with n ranging from 1 to 30, the study contemplated 1826 potential reactions encompassing bond dissociation and radical formation. In each of these reactions, the change in energy was determined by comparing the total energy of the reactants (the initial MAA molecules) against that of the products (resultant molecules post bond dissociation or radical formation).

The estimation of activation energy was conducted using transition state theory. This theory examines the behaviour of molecules during a chemical reaction, focusing on the highest potential energy point along the reaction pathway, known as the transition state. This state represents a critical energy barrier that the reactants have to surpass to transform into products. Characteristically, the transition state is a fleeting, high-energy arrangement of atoms, representing a peak on the reaction coordinate. To ascertain the transition state geometry, the study employed the functional thrust radius Newton–Raphson algorithm, tracing the collective chemical reaction coordinate. The accuracy of these obtained transition state geometries was then validated through the computation of their geometrical Hessian. This step involved confirming the appropriate curvature of the Potential Energy Surface (PES) at the transition state, ensuring it represented a saddle point on the PES—a maximum along the reaction coordinate and a minimum in all other dimensions.

The obtained data from quantum chemistry simulations were used to determine the fitting parameters X, F, and G (Table 1). The fitting procedure assumed samples were under irradiation of γ-rays (dose rate 20 cGy/min), and $P_{act}\left(U\right)$ and $P_{brk}\left(U\right)$ functions were selected according to kinetic MAA polymers growth models. The developed Monte Carlo model then used the effective events technique for MAA polymer growth, in which all polymer growth chemical reactions were subsumed into two main probabilistic events: activation of MAA polymer and breakdown of MAA polymer (Figure 4). The first step contained MAA monomer activation and radical propagation in MAA polymer, and, in our model, was described via the activation function $P_{act}\left(U\right)$:

(1) $$P_{act}\left(U\right)=X\left(1-F\right)+XFerf\left(GU\right),$$

where the activation function parameters *X*, *F*, and *G* were explicitly dependent on the length of the MAA polymer. The second step contained the MAA polymer break-down and deactivation steps in the growth of the MAA polymer or MAA polymer branching. This step was also described by the breakdown function $P_{brk}\left(U\right)$:

(2) $$P_{brk}\left(U\right)=X\left(1-F\right)+XFerf\left(GU^{2}\right).$$

### 4.3. Chemical Events Monte Carlo Model: Simulation Parameters

**Figure 5:**
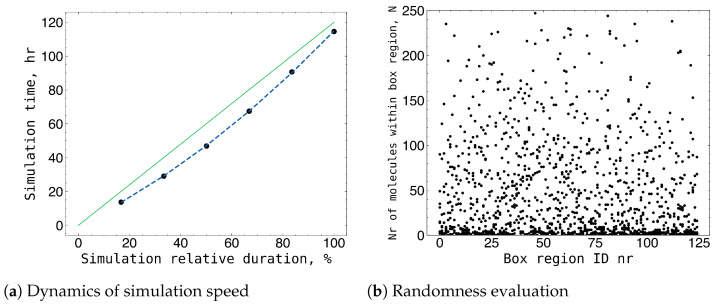
(**a**) Speed of the simulation for 50 nm × 50 nm × 50 nm box of default concentration (5875 molecules) varied from 1.3 h per million iteration in the beginning of the simulation and ended up to 1.9 h per million iteration. (**b**) An evaluation of the randomness of box structures based on the number of monomers in smaller box areas.

The iteration speed varied with the complexity of the structures being formed, commencing at 1.3 h per million iterations and increasing to 1.9 h per million iterations for a 50 nm × 50 nm × 50 nm box containing a default concentration of 5875 molecules (Figure 5a). Although box sizes up to 70 nm × 70 nm × 70 nm were simulated, the observed statistics remained largely unchanged for sizes exceeding 40 nm × 40 nm × 40 nm. Boxes measuring 30 nm × 30 nm × 30 nm yielded chemically infeasible results, indicating that the influence of box size on the properties at this scale became negligible beyond the minimum viable size of 40 nm × 40 nm × 40 nm. To evaluate the randomness of the structures created, the default size of the simulation box was divided into 125 smaller cubes measuring 10 nm × 10 nm × 10 nm, and the quantity of monomers in each cube was determined. The number of monomers determined for each cube was then compared to the number of monomers calculated for the equivalent cube in the other boxes. In total, 10 default-sized boxes were examined for structural similarities in this manner. The determined average deviation of 51.9 indicates that the produced structures in the various boxes had no correlation (Figure 5b).

The probability of chemical activation (connection) for methacrylic acid monomer was calculated in Equation (1). The probability of chemical break-down (division) for methacrylic acid monomer was calculated in Equation (2). The data for calculations of probabilities of chemical activation and break-down were taken from the quantum chemical computational model, which predicted the electronic structure of molecules using the hybrid B3LYP exchange-correlation functional and the def2-SVPD basis set (Table 1).

## Figures and Tables

**Table 1:** Data for calculations obtained from B3LYP/def2-SVPD model *. The X, F, and G parameters in the B3LYP/def2-SVPD model were the dimensionless coefficients calculated from the fitting procedure and used to define the likelihood of MAA monomer combination and growth or division and break down under γ-irradiation of 20 cGy/min dose rate.

Size of Polymer in Monomers	X	F	G
1	1.00	0.0	0.0
2	0.83	0.8	1.0
3	0.72	0.8	5.2
4	0.59	0.4	3.71
5	0.51	0.6	1.28
6–10	0.23	0.75	0.42
11–15	0.2	0.67	0.36
≥ 16	0.18	0.6	0.33

${}^{*}$ The parameters were calibrated using a probabilistic events model designed for MAA polymers, specifically for lengths comprising 1, 3, 5, 9, 13, and 18 monomer units (385 chemical reactions calculations at B3LYP/def2-SVPD level of theory).

## Data Availability

Draft source code of the simulation program is publicly available at the GitHub repository https://github.com/aleksandras-sevcik-edu/growpoli_alpha, accessed on 10 August 2023.

## Supplementary Materials

The following supporting information can be downloaded at: https://zenodo.org/records/10115953, accessed on 26 November 2023. Figure S1: Pseudo-code implemented for MC cycle. Figure S2: Pseudo-code for event handling. Figure S3: Simulation variables.
